# Progesterone decreases gut permeability through upregulating occludin expression in primary human gut tissues and Caco-2 cells

**DOI:** 10.1038/s41598-019-44448-0

**Published:** 2019-06-10

**Authors:** Zejun Zhou, Chuanxiu Bian, Zhenwu Luo, Constance Guille, Elizabeth Ogunrinde, Jiapeng Wu, Min Zhao, Sylvia Fitting, Diane L. Kamen, Jim C. Oates, Gary Gilkeson, Wei Jiang

**Affiliations:** 1 State Key Laboratory of Developmental Biology of Freshwater Fish, College of Life Sciences, Hunan Normal University, Changsha, 410081 China; 2 Department of Microbiology and Immunology, Medical University of South Carolina, Charleston, SC 29425 USA; 3 School of Medicine, Jiangsu University, Zhenjiang, Jiangsu 212013 China; 4 Department of Psychiatry and Behavioral Sciences, College of Medicine, Medical University of South Carolina, Charleston, SC 29425 USA; 5 Department of Obstetrics and Gynecology, Medical University of South Carolina, Charleston, South Carolina USA; 6 Biochemistry, Biophysics and Molecular Biology, Whitman College, Walla Walla, WA 99362 USA; 7 Department of Biochemistry, Basic Medical College, Hubei University of Chinese Medicine, Wuhan, 430065 China; 8 Department of Psychology & Neuroscience, University of North Carolina at Chapel Hill, Chapel Hill, NC 27599 USA; 9 Medical Research Service, Ralph H. Johnson VAMC, Medical University of South Carolina, Charleston, SC 29403 USA; 10 Division of Rheumatology, Department of Medicine, Medical University of South Carolina, Charleston, SC 29425 USA; 11 Division of Infectious Diseases, Department of Medicine, Medical University of South Carolina, Charleston, SC 29425 USA

**Keywords:** Translational immunology, Translational research

## Abstract

Progesterone plays a protective role in preventing inflammation and preterm delivery during pregnancy. However, the mechanism involved is unknown. Microbial product translocation from a permeable mucosa is demonstrated as a driver of inflammation. To study the mechanism of the protective role of progesterone during pregnancy, we investigated the effect of physiologic concentrations of progesterone on tight junction protein occludin expression and human gut permeability *in vitro* and systemic microbial translocation in pregnant women *in vivo*. Plasma bacterial lipopolysaccharide (LPS), a representative marker of *in vivo* systemic microbial translocation was measured. We found that plasma LPS levels were significantly decreased during 24 to 28 weeks of gestation compared to 8 to 12 weeks of gestation. Moreover, plasma LPS levels were negatively correlated with plasma progesterone levels but positively correlated with plasma tumor necrosis factor-alpha (TNF-α) levels at 8 to 12 weeks of gestation but not at 24 to 28 weeks of gestation. Progesterone treatment increased intestinal trans-epithelial electrical resistance (TEER) in primary human colon tissues and Caco-2 cells *in vitro* through upregulating tight junction protein occludin expression. Furthermore, progesterone exhibited an inhibitory effect on nuclear factor kappa B (NF-κB) activation following LPS stimulation in Caco-2 cells. These results reveal a novel mechanism that progesterone may play an important role in decreasing mucosal permeability, systemic microbial translocation, and inflammation during pregnancy.

## Introduction

Pregnancy is a period of considerable physiological adaptations^1^. During this process, adaptations in maternal systemic immunity are presumed to be responsible for successful reproduction. Of note, two theories of immune alteration are prevalent^2^. First, the theory of general immune suppression has been widely accepted. This suppression can reduce the likelihood of an antigen-specific response against the semi-allogeneic fetus during pregnancy^2,3^. The second theory is that an extensive T helper (Th) 2 bias promotes tolerance to the fetus during pregnancy^2,3^. Meanwhile, maternal immune responses to non-fetus foreign antigens are tightly controlled during all stages of pregnancy to avoid excessive and persistent inflammatory responses, which are associated with poor pregnant outcomes^4^. For example, plasma levels of certain pro-inflammatory cytokines, including TNF-α, chemokine (C-C motif) ligand 2 (CCL2), chemokine (C-X-C motif) ligand 10 (CXCL10) and Interleukin (IL)-18, are reduced during a healthy pregnancy *in vivo*, whereas increased plasma levels of pro-inflammatory cytokines and chemokines such as TNF-α, IL-1β, IL-6 and CXCL8 have been observed in preterm birth^5^.

Progesterone is the key sex hormone in maintaining pregnancy that has been shown to inhibit inflammation^6^. Serum levels of progesterone are elevated throughout the three trimesters during pregnancy, reaching the highest levels during the third trimester^7^. Progesterone is thought to be responsible for much of fetal tolerance during pregnancy^8^. Elevated progesterone during pregnancy inhibits the development of Th1 immune responses and promotes Th2 immune responses^9^. Progesterone was also found to suppress fetal T cell differentiation into inflammatory Th17 cells^10^. In autoimmune diseases, pregnant systemic lupus erythematosus (SLE) patients were found to have reduced levels of progesterone and higher incidence of preterm birth when compared with healthy pregnant controls^11^. In addition, progesterone inhibits toll-like receptors (TLRs)-mediated activation of interferon regulatory factors (IRFs) in plasmacytoid dendritic cells (pDCs), suggesting that progesterone may ameliorate the interferon signature and SLE disease activity^12^. Several studies have found that progesterone suppresses the inflammatory response in many animal models^13^. Interestingly, vitamin D was also shown to exert many physiological activities during the very early stages of gestation in perfect synchrony with progesterone, including an anti-inflammatory role during pregnancy^14,15^. Other studies have also shown that maternal obesity is associated with low first trimester serum progesterone, suggesting maternal obesity as a risk factor for low progesterone during pregnancy^16,17^.

Tight junctions are composed of occludin, claudins, zonula occludens (ZO)-1, -2, and -3, and provide a barrier function by governing the permeability of mucosa containing endothelial and epithelial cells^18^. The disruption of mucosal barriers results in an increase in microbial translocation and systemic immune activation^18^. Several diseases, including type 1 diabetes, multiple sclerosis and rheumatoid arthritis, are characterized by elevated intestinal permeability and increased bacterial product translocation to the systemic circulation, which favors autoimmunity^19,20^. For example, active inflammatory bowel disease (IBD) is characterized by a “leaky” gastrointestinal tract, leading to bacterial product translocation and inflammation^21^. However, progesterone has beneficial effects on IBD disease activity by improving gastrointestinal barrier function during pregnancy^22^. Furthermore, progesterone protects blood-brain barrier (BBB) function through increased expression of tight junction proteins (occludin, claudin-5, and ZO-1)^23^.

The aim of this study was to investigate the association and effect of the female sex hormone progesterone on gut permeability, microbial translocation, and inflammation during pregnancy. We found that plasma LPS levels were decreased at 24 to 28 weeks of gestation compared to 8 to 12 weeks of gestation. Plasma LPS levels negatively correlated with plasma levels of progesterone, but directly correlated with plasma levels of TNF-α at 8 to 12 weeks of gestation. Progesterone decreased NF-κB activation in response to LPS, and further decreased permeability in human primary colon tissues and Caco-2 cells through upregulating tight junction protein occludin expression *in vitro*. These results suggest that the increased levels of progesterone during pregnancy may contribute to decreases in mucosal permeability and consequently microbial translocation and systemic inflammation.

## Subjects and Methods

### Study subjects

This study was approved by the Institutional Review Board for Human Research (IRB#Pro00021511) at the Medical University of South Carolina (MUSC). All methods were performed in accordance with the relevant guidelines and regulations. Participants (Table 1) were pregnant women receiving routine obstetrical care and were recruited into the study during their first prenatal visits (8 to 12 weeks of gestation). Women were excluded if they were younger than 18 years of age, more than 12 weeks of gestation, or unable to provide informed consents. Blood samples were collected at 8 to 12 weeks of gestation (visit 1), 24 to 28 weeks of gestation (visit 2) and 6 to 8 weeks postpartum (as controls, visit 3) (Table 1).

**Table 1:** Clinical characteristics.

Characteristic	
Numbers of subjects	33
Gender	Female
Age (years) median*	28 (24–32)
Maternal BMI at visit 1 (kg/m^2^)*	29 (24–35)
Gravidity*	2 (1–4)
Parity*	1 (0–1)
Previous psychiatric disorders	No
Alcohol drinking at past 12 hours	No
Total gestation weeks*	39 (39–40)
Baby birth weight (oz)*	120 (107–130)
Fetal sex (%)	
Male	28
Female	72
Delivery type (%)	
Vaginal	80
Cesarean	20
Ethnicity (%)	
Caucasian	52
Africa American	45
Asian	3
Education (%)	
Less than high school+	6
High school	39
Some college	37
College or higher	18
Family income (%)	
Less than $20,000+	15
$20,000–$49,999	40
$50,000–$74,999	33
$75,000 or more	12
Gestational diabetes mellitus	2
Preeclampsia	0
Hormone replacement therapies	No
Systemic healthy and no smokers	Yes
Systemic antibiotic treatment (past 6 months)	No

BMI: body mass index.

*Data are median (interquartile range) values.

### Plasma sample preparations

Plasma was isolated by centrifugation of EDTA-contained blood, aliquoted, and stored at −80 °C until they were thawed for analysis.

### Plasma levels of LPS

Plasma samples were diluted to 10% with endotoxin-free water and were heated to 80 °C for 10 minutes to inactivate inhibitory proteins in plasma. LPS levels in plasma were then quantified using the limulus amebocyte lysate QCL-1000 kit (Lonza, Walkersville, MD, USA) as described in our previous studies^24–26^.

### Plasma levels of progesterone, 17β estradiol, estriol, TNF-α, IL-6, IL-1β, and vitamin D

Plasma levels of progesterone, estradiol and estriol were determined using ELISA kits (catalog numbers: ab108670, ab108667, and ab108671; Abcam, Cambridge, MA, USA) according to the manufacturer’s instructions. Plasma levels of TNF-α, IL-6 and IL-1β were measured using high-sensitivity ELISA kits (catalog numbers: HSTA00E, HS600B, and HSLB00D; R&D, Inc, Minneapolis, MN, USA) according to the manufacturer’s instructions. A rapid, direct RIA developed in the Hollis laboratory and manufactured by Diasorin Corporation (Stillwater, MN) was used to measure total circulating 25(OH)D concentration in plasma samples as previously described^27,28^.

### Immunohistochemical analysis of occludin

Fresh human colon biopsies from non-cancerous tissues of postmenopausal females were obtained from Hollings Cancer Center, a biorepository at MUSC. Gelfoam (Gelfoam absorbable gelatin sponge, Pfizer Inc., NY, USA) was distributed into 12-well tissue culture plates. Gut samples from healthy postmenopausal females including 3 Caucasians and 3 African Americans (3 mm × 3 mm) were placed on the gelfoam and cultured at 37 °C with phenol red-free RPMI-1640 media (Gibco, Waltham, MA, USA) containing penicillin (100 unit/mL), streptomycin (100 µg/mL) and 10% charcoal-stripped FBS (Gibco). The tissues were treated with media or different concentrations (20 and 125 ng/mL, dissolved in ethanol to a stock concentration of 1 mg/mL, with further dilutions made in medium) of progesterone (catalog number: P0130, Sigma, St Louis, MO, USA) for 24 h. Tissue samples were then harvested, fixed in formalin, embedded in paraffin and subsequently stained by immunohistochemical techniques^29^. For occludin detection, rabbit anti-human occludin antibody in a 1:250 dilution in 3% BSA (catalog number: 40-4700, Invitrogen, CA, USA) was added to the slide. The slide was incubated at 4 °C overnight. After washing three times with TBST, horseradish peroxidase-conjugated goat anti-rabbit IgG in a 1:1000 dilution (KPL) were added to the slide. The slide was incubated at 37 °C for 1 h. The slide was washed three times with TBST. The color was developed using the DAB substrate kit (BD Biosciences, San Jose, CA, USA). The slide was observed with a fluorescent microscope (Zeiss Axio Vet. A1). The experiment was performed three times. The slide was stained with the rabbit anti-human occludin antibody alone or was stained with horseradish peroxidase-conjugated goat anti-rabbit IgG alone set up as controls.

### Quantitative real time reverse transcription-PCR (qRT-PCR) analysis of occludin mRNA expression

Gut tissue samples were treated as above, and then extracted for total RNA by EZNA Total RNA Kit (Omega Bio-tek, Doraville, GA, USA). cDNA was synthesized from 5 μg of RNA from each sample using the SuperScript III First-Strand Synthesis System (Invitrogen) followed by PCR. qRT-PCR was carried out in a thermal cycler (C1000™ Thermal Cycler, BioRad, Hercules, CA, USA) using the PerfeCTa SYBR Green SuperMix (Quanta Biosciences, Gaithersburg, MD, USA). Primer sequences, reaction conditions, and optimal cycle numbers of PCR were described previously^30^. The expression level of occludin was analyzed using the comparative threshold cycle method (2^−ΔΔCt^) with GAPDH as an internal reference^29,30^. The experiment was performed three times.

### Caco-2 cell culture

The Caco-2 cells, a human epithelial colorectal cancer cell line, were purchased from ATCC (Manassas, VA, USA). Caco-2 cells were maintained in Dulbecco’s Modified Eagle’s medium (DMEM, phenol red-free, Thermo Fisher Scientific, Waltham, MA, USA) supplemented with 20% charcoal-stripped FBS, 2 mM L-glutamine, 10 mM HEPES, 100 unit/mL penicillin and 100 µg/mL streptomycin (Gibco) in a humidified 37 °C, 5% CO2 incubator. Cells were grown in 25 cm² rectangular canted neck cell culture flask with vent cap (Corning, Corning City, NY, USA). The medium was changed every three days. The cells were harvested with 0.25% trypsin-EDTA (Gibco).

### Immunofluorescence analysis of occludin and p65

Caco-2 cells were cultured as above and grown in 6-well plates. Cells were used for analysis between days 14 and 21. The cells were treated with media alone (control), or different concentrations (20 and 125 ng/mL) of progesterone in the presence or absence of 1 μM mifepristone (progesterone receptor antagonist, catalog number: M8046, Sigma, dissolved in ethanol to a stock concentration of 1 mM, with further dilutions made in medium) for 24 h. After stimulation, cells were washed with TBST and incubated with 4% paraformaldehyde for 15 min. Cells were then blocked with 5% BSA for 2 h followed by incubation overnight with rabbit anti-human occludin antibody in a 1:400 dilution. After washing three times with TBST, DyLight 594 labeled goat anti-rabbit second antibody in a 1:1000 dilution (KPL) was added to the cells and incubated at 37 °C for 1 h. After 1 h, the slide was washed three times with TBST and covered with mounting medium (Vectashield Hard-Set Mounting Medium with DAPI; catalog number: H-1500, Vector Labs., Burlingame, CA, USA). The slide was observed with a fluorescent microscope. The experiment was performed three times.

For p65 detection, Caco-2 cells were cultured as above and then treated with media alone (control), or different concentrations of progesterone (20 and 125 ng/mL) in the presence or absence of 1 μM mifepristone 24 h, followed by stimulation with 200 ng/mL of LPS (catalog number: L4524, Sigma, dissolved in PBS) for 30 minutes. After stimulation, cells were washed with TBST and incubated with 4% paraformaldehyde for 15 min. After incubation, cells were permeabilized with 0.1% Triton X-100 for 5 min. The slide was washed three times with TBST, and blocked with 5% BSA for 2 h followed by incubation overnight with primary antibody rabbit monoclonal p65 in a 1:400 dilution (catalog number: 8242P, Cell Signaling, Beverly, MA, USA). The experiment was performed three times.

### Fluorescence intensity analysis

Occludin images were taken with the 40X objective using a red fluorescence filter. All images were taken using an identical camera, microscope lens, and light settings. Fluorescence intensity analysis was performed using ImageJ software (ImageJ, Bethesda, USA) as described previously^29^. Briefly, each image was inverted to gray-scales and a region of interest from the image was drawn at first. Next, images were calibrated by the optical density, and then integrated densities were measured. The same method was used for analyzing the background intensity of no cell regions. After background subtraction, the fluorescence intensity was calculated as integrated fluorescence intensity per micrometer of area.

### Western blot analysis of occludin, ZO-1, Claudin-1 and p65

Caco-2 cells were cultured as above with media alone (control), or different concentrations (20 and 125 ng/mL) of progesterone in the presence or absence of 1 μM mifepristone for 24 h. After stimulation, cells were harvested and lysed in ice-cold RIPA buffer (Alfa Aesar, Reston, VA, USA). 40 μg of the whole-cell extracts were loaded in each lane on a 10% SDS-PAGE gel and then transferred onto a PVDF membrane (Invitrogen). For occludin detection, the membrane was blocked with TBST containing 5% BSA for 2 h, and incubated with rabbit anti-human occludin antibody in a 1:400 dilution at 4 °C overnight. For ZO-1 and Claudin-1 detection, the ZO-1 (1:500 dilution, catalog number: PA5-21965, Invitrogen) and Claudin-1 (1:1000 dilution, catalog number: 51-9000, Invitrogen) antibodies were used. The rabbit anti-β-actin antibody in a 1:1000 dilution (Santa Cruz, CA, USA) was employed as a negative control. After washing with TBST three times, the membranes were incubated with horseradish peroxidase-conjugated goat anti-rabbit IgG in a 1:1000 dilution (KPL) for 1 h, followed by thorough washing. The target proteins were developed using the DAB substrate kit (BD Biosciences). The experiment was performed three times.

For p65 phosphorylation detection, Caco-2 cells were treated with media alone (control), or different concentrations (20 and 125 ng/mL) of progesterone in the presence or absence of 1 μM mifepristone for 24 h, followed by stimulation with 200 ng/mL of LPS for 15 min. After stimulation, cells were harvested and lysed in ice-cold RIPA buffer containing protease and phosphatase inhibitors (catalog number: 1861281, Thermo Scientific). After blotting, the membrane was blocked with TBST containing 5% BSA for 2 h, and incubated with primary antibody rabbit monoclonal p65 in a 1:400 dilution or rabbit anti-human p-p65 antibody in a 1:400 dilution (catalog number: 3033P, Cell Signaling) at 4 °C overnight. The experiment was performed three times.

### Trans-epithelial electrical resistance (TEER)

Caco-2 cells were grown in 24-well plates and seeded at 20,000 cells per insert on 6.5 mm diameter and 0.4 mm pore PET membrane insert (Corning). The medium was changed every three day. Cells were used for experiment between days 14 and 21. Caco-2 cells were treated with media alone (control), or different concentrations (20 and 125 ng/mL) of progesterone in the presence or absence of 1 μM mifepristone for 24 h. After 24 h, confluent monolayers were washed three times with pre-warmed PBS, left for 30 min at 37 °C to equilibrate, and then TEER was measured using the Millicell-Electrical Resistance System (ERS, Millipore, Billerica, MA, USA). The experiment was performed three times.

### Analysis of TEER and Occludin expression after plasma treatment

Caco-2 cells were grown in 24-well plates and seeded at 20,000 cells per insert as above. Caco-2 cells were treated with media alone or 200 μL plasma from 4 normal pregnant women (at visit 2) in the presence or absence of 1 μM mifepristone for 24 h. TEER and Occludin mRNA expression were detected as above.

### Statistical analysis

Data from repeated experiments were shown as mean and standard error (SEM). Statistical analysis was performed by GraphPad Prism 6.0 (GraphPad, San Diego, USA) using the One-way ANOVA and Spearman correlation test. P values of ≤0.05 were considered statistically significant.

### Importance

Increased progesterone may play an important role in healthy maternal immune status during pregnancy through decreasing mucosal permeability, systemic microbial product translocation, and inflammation.

## Results

### Decreased plasma levels of LPS in healthy pregnant women during 24 to 28 weeks of gestation

**Figure 1:**
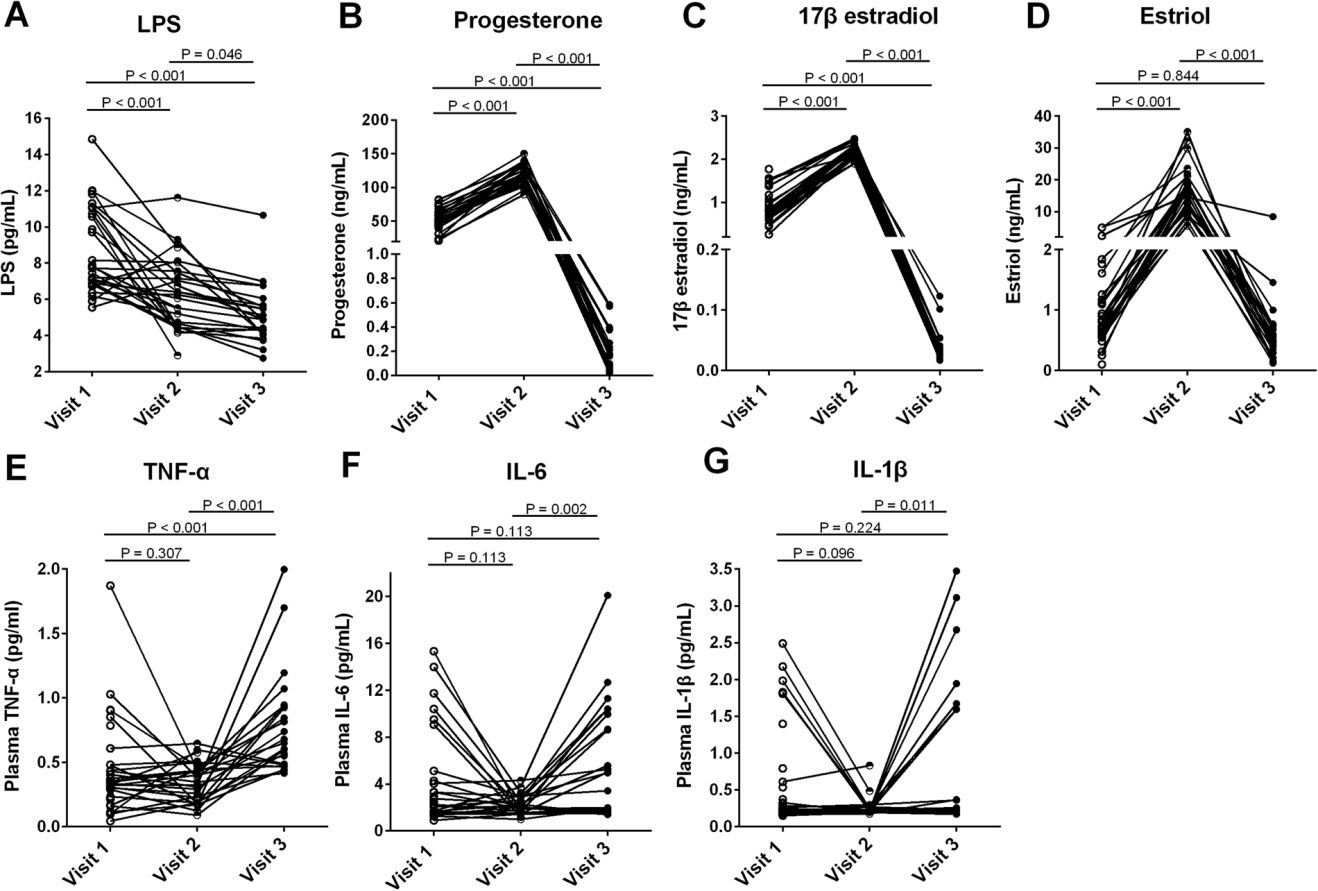
Plasma levels of LPS, progesterone, 17β estradiol, estriol, TNF-α, IL-6 and IL-1β at 8 to 12 weeks of gestation (visit 1), 24 to 28 weeks of gestation (visit 2) and 6 to 8 weeks postpartum (visit 3) in healthy pregnant women. Plasma levels of LPS (**A**) were analyzed using the limulus amebocyte lysate assay. Plasma levels of progesterone (**B**), 17β estradiol (**C**), and estriol (**D**), and plasma levels of TNF-α (**E**), IL-6 (**F**) and IL-1β (**G**) were analyzed using ELISA assays. The data were shown as median. One-way ANOVA.

In order to assess systemic microbial translocation, plasma levels of LPS from 33 healthy pregnant women were analyzed at 8 to 12 weeks of gestation (visit 1) and 24 to 28 weeks of gestation (visit 2). Compared to visit 1, the plasma LPS levels were significantly decreased at visit 2 (P < 0.001, Fig. 1A). The median plasma levels of LPS (pg/mL) were 7.76 (6.86–11.04) and 6.25 (4.53–7.55) at visit 1 and visit 2 respectively. Moreover, plasma levels of LPS at 6 to 8 weeks postpartum (controls, visit 3) were also significantly decreased. The median plasma levels of LPS (pg/mL) at visit 3 were 4.88 (4.10–5.66). In addition, there was no difference in plasma levels of 25(OH)D at visits 1, 2 and 3 (Supplemental Fig. 1A).

### Increased plasma levels of progesterone, 17β estradiol and estriol in healthy pregnant women during 24 to 28 weeks of gestation

Next, plasma levels of female sex hormones were evaluated. Compared to visit 1, the plasma levels of progesterone, 17β estradiol and estriol were significantly increased at visit 2 (P < 0.001, Fig. 1B–D). The median concentrations were 52.14 (44.51–61.76) and 120.10 (111.00–133.80) for progesterone (ng/mL), 0.84 (0.66–1.40) and 2.22 (2.09–2.33) for 17β estradiol (ng/mL), and 0.74 (0.59–1.50) and 16.95 (11.47–19.43) for estriol (ng/mL) at visit 1 and visit 2 respectively. However, the plasma levels of progesterone, 17β estradiol and estriol at visit 3 (6 to 8 weeks postpartum) were significantly reduced compared to those at visit 2. The median concentrations of progesterone, 17β estradiol and estriol at visit 3 (ng/mL) were 0.10 (0.07–0.27), 0.03 (0.02–0.04) and 0.51 (0.34–0.74) respectively.

### Pro-inflammatory cytokine levels

TLR4 cell signaling downstream pro-inflammatory cytokines were measured during pregnancy^31^. There was no difference in plasma levels of TNF-α, IL-6 and IL-1β at visits 1 and 2 (Fig. 1E–G). The median concentrations were 0.33 (0.22–0.48) and 0.35 (0.19–0.44) for TNF-α (pg/mL), 2.08 (1.47–4.16) and 1.98 (1.49–2.61) for IL-6 (pg/mL), and 0.24 (0.20–0.70) and 0.22 (0.20–0.25) for IL-1β (pg/mL) at visit 1 and visit 2 respectively. However, the plasma levels of TNF-α, IL-6 and IL-1β at visit 3 (6 to 8 weeks postpartum) were significantly increased compared to those at visit 2. The median concentrations of TNF-α, IL-6 and IL-1β at visit 3 (pg/mL) were 0.65 (0.48–0.94), 4.95 (1.76–9.96) and 0.25 (0.22–1.60) respectively.

### Correlation between plasma levels of LPS, sex hormones, and cytokines

**Figure 2:**
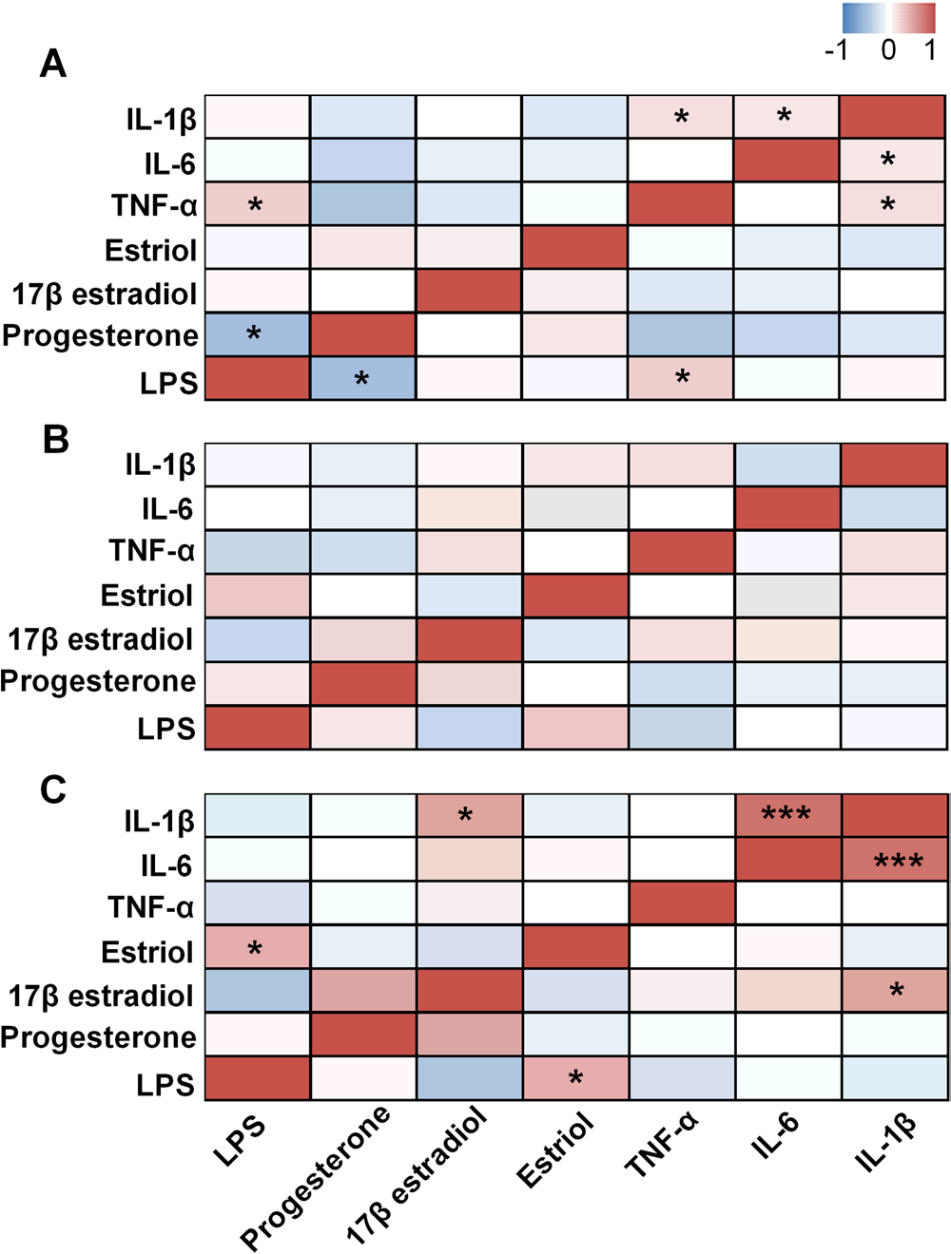
Correlations of plasma LPS, progesterone and TNF-α at 8 to 12 weeks of gestation but not at 24 to 28 weeks of gestation and 6 to 8 weeks postpartum. In the heat map, asterisks denote correlations between LPS, progesterone, 17β estradiol, estriol, TNF-α, IL-6 and IL-1β at 8 to 12 weeks of gestation (visit 1, **A**), 24 to 28 weeks of gestation (visit 2, **B**) and 6 to 8 weeks postpartum (visit 3, **C**) in healthy pregnant women. *P < 0.05; ***P < 0.001. Spearman correlation test.

To further investigate the interactions between LPS and sex hormones, we analyzed the correlations between the plasma levels of LPS and sex hormones. A negative relationship was observed between the plasma LPS and progesterone levels at visit 1 but not at visit 2 and 3 (Fig. 2). Moreover, a direct correlation was observed between plasma LPS levels and plasma levels of TNF-α at visit 1 but not at visit 2 and 3 (Fig. 2). Notably, there was a marginally inverse correlation between progesterone and TNF-α at visit 1 (r = − 0.30, P = 0.08) but not at visit 2 to 3. However, there were no significant correlations between the plasma levels of LPS and other cytokines (IL-6 and IL-1β), nor between progesterone levels and other cytokines (IL-6 and IL-1β). There were also no significant correlations between the plasma levels of LPS, estradiol, estriol and cytokines (TNF-α, IL-6 and IL-1β). As maternal vitamin D status is one of the important predictors of pregnancy outcomes (preterm delivery), therefore, we further analyzed the correlations between progesterone and LPS levels in different subgroups based on the plasma vitamin D concentration (<20 ng/mL is severe deficiency; 20 ng/mL–40 ng/mL is standard deficiency; >40 ng/mL is a normal level)^27,28^. We found no significant correlations between progesterone and LPS levels in the different vitamin D subgroups (Supplemental Fig. 1B). We also found there are no significant correlations between vitamin D and progesterone, LPS levels, gestation weeks and cytokines (TNF-α, IL-6 and IL-1β) (Supplemental Fig. 1B). Since evidence has shown that perturbation of the intestinal microbiota and changes in intestinal permeability will trigger inflammation in obese mothers^17,32^, we further analyzed the plasma levels of LPS, progesterone, 17β estradiol, estriol, cytokines (TNF-α, IL-6 and IL-1β) and vitamin D levels based on the BMI value. However, we did not find any significant difference in these two groups (BMI < 30 and BMI ≥ 30) (Supplemental Fig. 2).

### Increased occludin expression and decreased gut permeability by progesterone treatment in primary gut tissues and Caco-2 cells

**Figure 3:**
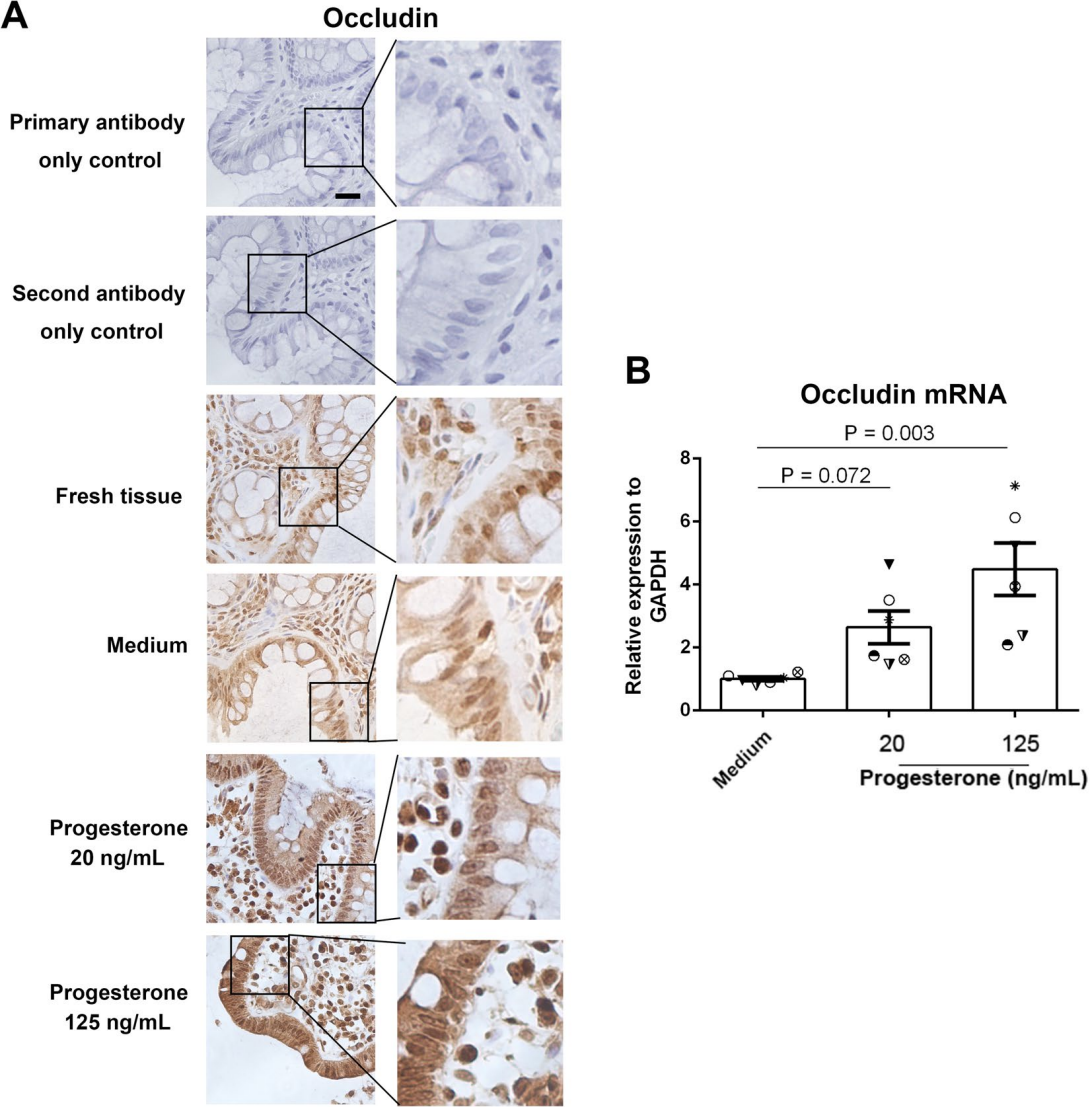
Effect of progesterone on occludin expression in human primary gut tissues. Female primary gut tissues were treated with media alone or different concentrations of progesterone for 24 h. Occludin expression was then examined by immunohistochemistry assay (**A**). Data are representative of at least three independent experiments. Bar: 20 μm. Occludin expression was examined by qRT-PCR in female primary gut tissues treated by different conditions (**B**). One-way ANOVA.

To further investigate the role of progesterone on human gut permeability, we analyzed the expression of occludin, a tight junction protein, in primary human gut tissues. Female primary gut tissues were treated with media alone or different concentrations of progesterone, including physiologic concentrations of progesterone during pregnancy and non-pregnancy in women^7^. Occludin expression was then examined by immunohistochemistry. Increased occludin expression was observed in gut tissues following progesterone treatment *in vitro* (Fig. 3A). Furthermore, the occludin mRNA expression in these treated primary gut tissues were analyzed by qRT-PCR. Consistently, progesterone significantly increased occludin mRNA expression in a dose-dependent manner (Fig. 3B).

**Figure 4:**
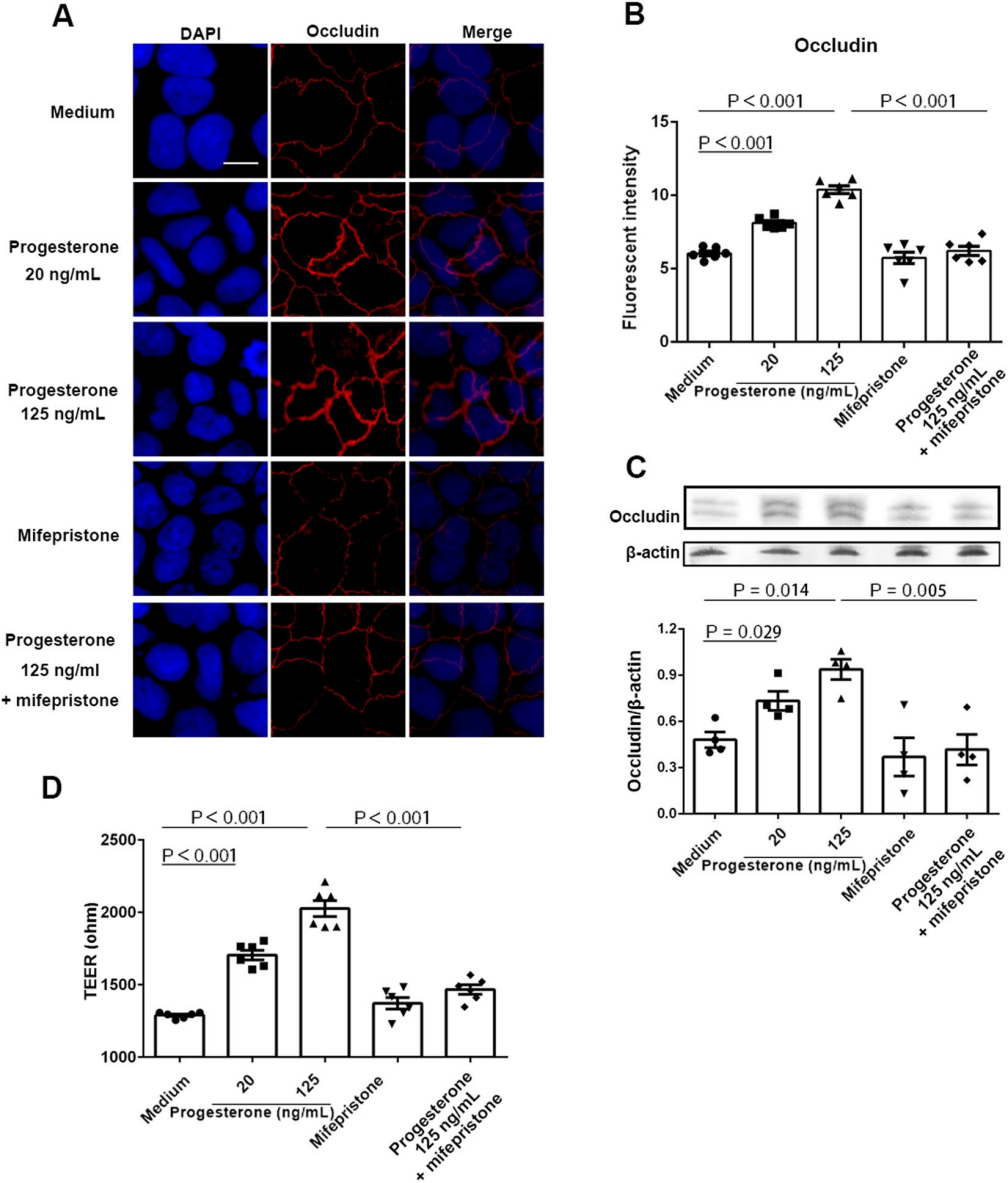
Effect of progesterone on Caco-2 cells permeability. Caco-2 cells were treated with media alone or different concentrations of progesterone in the presence or absence of 1 μM mifepristone for 24 h. Occludin expression was then examined by immunofluorescence assay (**A**). Occludin and nuclei were detected by DyLight 594-labeled antibody and DAPI respectively. Data are representative of at least three independent experiments. Bar: 20 μm. Images were taken with a fluorescence microscope, and fluorescence intensity of occludin was analyzed using ImageJ software (**B**). Occludin expression in Caco-2 cells was examined by western blot (**C**). Relative expression of occludin was quantified to β-actin by ImageJ software. Trans-epithelial electrical resistance (TEER) was measured in Caco-2 cells (**D**). Data are representative of at least three independent experiments. One-way ANOVA.

To further verify the effect of progesterone on occludin expression, Caco-2 cells were treated with progesterone and examined for occludin expression by immunofluorescence analysis. Consistently, dose-dependent increases in occludin expression were observed in Caco-2 cells following progesterone treatment *in vitro* (Fig. 4A,B and Supplemental Fig. 4). To examine the specificity, a progesterone inhibitor mifepristone^33^ was used and the increase of occludin mRNA expression by progesterone was abrogated when Caco-2 cells were cultured with mifepristone plus progesterone. Moreover, occludin protein expression was increased by progesterone using western blot (Fig. 4C and Supplemental Fig. 5). However, there were no significant differences in ZO-1 and Claudin-1 expression (Supplemental Fig. 3). Notably, occludin is a tight junction protein. To investigate the functional effect of occludin upregulation by progesterone, we evaluated Caco-2 cell permeability by TEER. We found that the TEER values were significantly increased following treatment with progesterone (Fig. 4D).

### Inhibitory effect of progesterone on LPS-induced NF-κB activation

**Figure 5:**
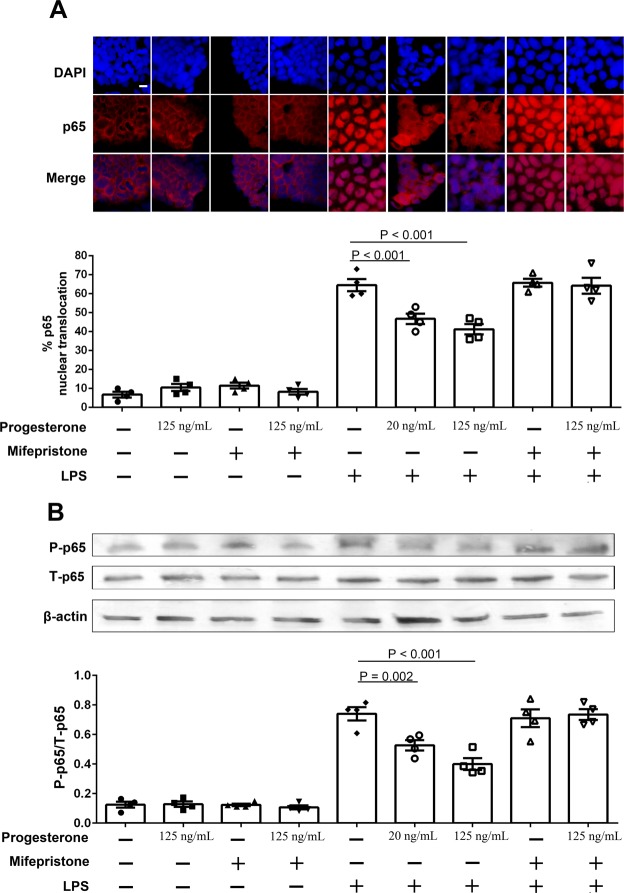
Effects of progesterone on the NF-κB signaling pathway in Caco-2 cells. Caco-2 cells were pretreated with media alone or different concentrations of progesterone in the presence or absence of 1 μM mifepristone for 24 h, followed by 30 minutes stimulation with 200 ng/mL of LPS. p65 was detected by immunofluorescence assay (**A**). p65 and nuclei were stained with DyLight 594-labeled antibody and DAPI respectively. Bar: 20 μm. Percentages of p65 nuclear translocation were determined by counting 100 cells in 3 or 4 non-overlapping fields. p65 and phospho-p65 (P-p65) were examined by western blot in Caco-2 cells after LPS (200 ng/mL) stimulation for 15 minutes (**B**). Relative expression of P-p65 was quantified to total-p65 (T-p65) by ImageJ software. Data are representative of at least three independent experiments. One-way ANOVA.

To investigate the effect of progesterone on gut epithelial cell activation, we stimulated Caco-2 cells with LPS in the presence or absence of progesterone. Stimulation of Caco-2 cells with LPS alone increased the translocation of NF-κB p65 into the nucleus as shown by immunofluorescence staining of p65 (Fig. 5A). In contrast, pre-treatment with progesterone inhibited the translocation of p65 (Fig. 5A). Moreover, the inhibitory effect of progesterone on p65 translocation was directly blocked by progesterone plus mifepristone in Caco-2 cells (Fig. 5A). Notably, phosphorylation levels of p65 were increased in Caco-2 cells treated with LPS alone (Fig. 5B and Supplemental Fig. 6), and treatment of progesterone inhibited LPS-mediated p65 phosphorylation but was reversed by mifepristone (Fig. 5B). The inhibitory effect of progesterone on LPS-mediated phosphorylation of p65 was in a dose-dependent manner. These results indicate progesterone prevents LPS-induced NF-κB activation in Caco-2 cells.

## Discussion

LPS, a representative biological marker of systemic microbial translocation, induces the production of pro-inflammatory cytokines and activation of NF-κB, leading to immune activation^34^. Importantly, inflammation mediated by the maternal immune system is necessary for embryo implantation, placentation, and parturition during pregnancy^35^. Recently, higher levels of microbial translocation were observed in HIV-infected pregnant women and were associated with preterm delivery^36^. In the present study, we showed that pregnant mothers at 24 to 28 weeks of gestation exhibited significantly reduced plasma levels of LPS compared to those at 8 to 12 weeks of gestation, suggesting that microbial translocation is probably inhibited in this phase. A previous study has investigated the plasma levels of progesterone, estradiol and estriol during pregnancy^7^. Consistent with this study, we also found that the plasma levels of progesterone, 17β estradiol and estriol were significantly higher at 24 to 28 weeks of gestation compared to those at 8 to 12 weeks of gestation. Graham *et al*. have reported that most pro-inflammatory cytokine levels were reduced during pregnancy compared to those in post-pregnancy *in vivo*^5^. Similarly, our findings showed that the plasma levels of TNF-α, IL-6 and IL-1β at 24 to 28 weeks of gestation were significantly reduced compared to 6 to 8 weeks postpartum.

Previous studies suggest that an anti-inflammatory response is necessary for fetal growth at the second trimester as any ongoing pro-inflammatory signals and infection at this stage can lead to miscarriage or preterm birth^37,38^. Consistent with these results, we found decreased LPS levels at the second trimester compared to the first trimester. Nonetheless, we propose that inflammation may play a double-edged sword role during pregnancy^39^. On the one hand, a pro-inflammatory stage is responsible for the implantation, placentation and parturition^37^. On the other hand, a previous study suggested that viral infection of the placenta during pregnancy can elicit a higher fetal inflammatory response that, in turn, can cause organ damage and potentially downstream developmental deficiencies^40^. Higher levels of microbial translocation were also observed in HIV-infected pregnant women and were associated with preterm delivery^36^. Interestingly, we also found decreased LPS levels at 6 to 8 weeks postpartum in our study. This may be due to the presence of increased anti-LPS antibodies after the baby is delivered, but further experiments are needed to clarify the effect.

As previously reported, progesterone plays a critical role in anti-inflammatory responses and immune suppression during pregnancy^8^. Low progesterone level is a known risk factor for preterm birth in pregnancy^41^. Indeed, we found an inverse correlation of progesterone and plasma LPS at 8 to 12 weeks of gestation, suggesting that progesterone may inhibit plasma LPS levels and likely its associated inflammation. This is consistent with a previous study showing that plasma soluble CD14 (a marker of monocyte activation in response to LPS stimulation) at the first trimester but not at the later period of pregnancy predicts preterm delivery^36^. In addition, plasma TNF-α levels in early pregnancy are higher in patients with recurrent miscarriage compared to normal pregnant controls^42^. Vitamin D is essential for the health of both mother and fetus during pregnancy^43^. However, no significant correlations between vitamin D and gestation weeks, progesterone, LPS levels, 17β-estradiol, estriol and cytokines were found in our study. Maternal obesity is also one of the factors that can influence progesterone levels during pregnancy^16,17,32^. However, we did not find any significant difference in these two subgroups (BMI < 30 and BMI ≥ 30). Hence, it is possible that progesterone may be mediated by multiple factors, such as vitamin D, obesity, race, offspring birth-weight, bone mass, serum calcium concentrations and so on, through an unknown mechanism^16,17,32,43,44^.

It is now recognized that fetal tolerance during pregnancy is maintained through a shift in the pattern of cytokine production from a “Th1 type” profile toward more of a Th2 pattern^45^. A previous study showed that treatment with 10 μM progesterone decreased TNF-α production to maternal T cells *in vitro*, suggesting that decreased TNF-α by progesterone is important in fetal tolerance^8^. However, IL-10, which would classically be described as a Th2 cytokine, is also significantly decreased following treatment with 10 μM progesterone^8^. IL-10 is an anti-inflammatory cytokine that can suppress the production of inflammatory cytokines such as IL-6 and IL-1β^46^. In the current study, we found plasma LPS was directly correlated with the pro-inflammatory cytokine TNF-α, but not IL-6 and IL-1β at 8 to 12 weeks of gestation. It can be speculated that the ratio of TNF-α/IL-10 production during pregnancy may have an influence on maternal inflammation and its associated cytokines production.

Estradiol and estriol are two key pregnancy-associated hormones^47^. In addition, estrogen may have an effect on mucosal permeability, and subsequently ignite elevated microbial translocation and systemic inflammation^29,48^. In this study, the increased ratios of estradiol/progesterone and estriol/progesterone at visit 2 and 3 may be the reason why we found no significant correlations between LPS, TNF-α and progesterone in these two visits. Moreover, in a previous study, first trimester sCD14 levels independently associated with preterm delivery among HIV-infected women^36^. The correlations of first trimester suggest a basal risk of inflammation in early pregnancy. In addition, we did not find any significant difference in TEER and occludin expression in Caco-2 cells following plasma treatment. This may also be because of the increased ratios of estradiol/progesterone and estriol/progesterone during pregnancy. Intriguingly, we also found a significant direct correlation between estradiol and IL-1β at 6 to 8 weeks postpartum. Altogether, our results indicate that the production of pro-inflammatory cytokines might be impacted through an indirect progesterone-mediated mechanism, in which elevated progesterone levels during pregnancy may prevent systemic microbial translocation through some unknown mechanisms and in turn restrict its-associated systemic inflammation. However, we cannot exclude that a direct anti-inflammatory effect of progesterone may also play a role in controlling systemic inflammation during pregnancy.

Tight junctions are essential for establishing a barrier in epithelial tissues and for defense against the invading pathogens^18^. Previous reports showed that tight junctions can be disrupted by several pathogens, which may result in increased mucosal permeability and subsequent systemic inflammation^18^. In the intestine, barrier loss can also be mediated by cytokines and epithelial myosin light chain kinase (MLCK) activation^49^. For example, TNF-α, which is critical to Crohn’s disease pathogenesis, impairs gut barrier integrity via MLC phosphorylation and occludin internalization^49–51^. However, TNF-α did not affect the internalization of ZO-1 and Claudin-1^49–51^. This suggests that pro-inflammatory cytokines may correlate directly with barrier impairment. Progesterone is known to have protective effects on barrier function and reduction of pro-inflammatory cytokines^52^. Pazos *et al*. have also reported that there is a strengthened immune barrier function at late pregnancy^3^. Similarly, we found increased plasma progesterone levels and decreased plasma LPS and pro-inflammatory cytokines levels at late pregnancy compared to early pregnancy in healthy pregnant women, suggesting that these events are closely linked. In addition, it has been shown that the level of occludin expression in epithelial cells was inversely correlated with paracellular permeability^53–55^. Moreover, administration of progesterone improved brain function through reducing BBB permeability and increasing occludin expression in rats with traumatic brain injury, and progesterone receptor antagonist directly inhibited these functions^23,56^. Braniste *et al*. previously found no significant effect of progesterone on colon epithelial cell permeability using chamber experiments in rat with bilateral ovariectomy^57^. This may result from the absence of overall progesterone receptor expression after bilateral ovariectomy^58^. Consistently, we observed that treatment with physiological concentrations of progesterone increased the expression of the tight junction protein occludin and decreased gut permeability in this study. In our results, ZO-1 and claudin-1 expression did not change after progesterone treatment. Under pathological conditions, alterations of ZO-1 and claudin-1 expression are often observed with blood-brain barrier breakdown^59^. In our study, treatment with physiologic concentrations of progesterone may account for the difference seen between others and ours. We do not know the detailed mechanisms for this difference, but we are planning future studies to investigate it. Taken together, these findings reinforce the hypothesis that progesterone may inhibit gut permeability and subsequent systemic microbial translocation during pregnancy, therefore, avoiding excessive and persistent inflammatory responses, which are associated with poor pregnancy outcomes.

Previous studies and our recent finding suggest that estradiol inhibits inflammatory responses^29,60^. Interestingly, a growing amount of literature shows that progesterone also has an anti-inflammatory effect in animal models through decreasing pro-inflammatory cytokines or suppressing NF-κB and MAPK activation^33,61^. Consistently, we found that progesterone prevented LPS-induced NF-κB activation in human gut epithelial cells. Hence, we propose that the progesterone-mediated inhibition of NF-κB may reduce the risk of excessive inflammatory responses during pregnancy.

In conclusion, this study demonstrates that microbial translocation is significantly decreased in pregnant women during 24 to 28 weeks of gestation compared to 8 to 12 weeks of gestation. Also, progesterone exhibits the ability to increase occludin expression and TEER values of Caco-2 cells, and the ability to inhibit LPS-induced NF-κB activation. The limitation of this study is the lack of samples from non-pregnant controls, which may account for similar levels of TNF-α, IL-6 and IL-1β in the two visits. These results provide new insights into the mechanism of progesterone on gut permeability and maternal inflammation.

## Supplementary information

supplemental material
