# Supplementary material for: Progesterone decreases gut permeability through upregulating occludin expression in primary human gut tissues and Caco-2 cells
Source: Sci Rep. 2019 Jun 10;9:8367. doi: 10.1038/s41598-019-44448-0 (PMC6558054; doi:10.1038/s41598-019-44448-0)
Supplement: Supplementary file 1 — supplemental material [file 41598_2019_44448_MOESM1_ESM.doc]

**Supplementary material**

**Progesterone decreases gut permeability through upregulating occludin expression in primary human gut tissues and Caco-2 cells**

Running title: Effect of progesterone on gut permeability

Zejun Zhou^a,b^, Chuanxiu Bian^b, c^, Zhenwu Luo^b^, Constance Guille^d^, Elizabeth Ogunrinde^b^, Jiapeng Wu^e^, Min Zhao^b,f^ , Sylvia Fitting^g^, Diane L. Kamen^h^, Jim C. Oates^h^, Gary Gilkeson^h^, *, Wei Jiang^b, i^, *

^a^State Key Laboratory of Developmental Biology of Freshwater Fish, College of Life Sciences, Hunan Normal University, Changsha, China, 410081

^b^Department of Microbiology and Immunology, Medical University of South Carolina, Charleston, SC, USA, 29425

^c^School of Medicine, Jiangsu University, Zhenjiang, Jiangsu, China, 212013

^d^Department of Psychiatry and Behavioral Sciences, College of Medicine, Medical University of South Carolina, Charleston, SC, USA, 29425

^e^Biochemistry, Biophysics and Molecular Biology, Whitman College, Walla Walla, WA, 99362

^f^Department of Biochemistry, Basic Medical College, Hubei University of Chinese Medicine, Wuhan, China, 430065

^g^Department of Psychology & Neuroscience, University of North Carolina at Chapel Hill, Chapel Hill, NC, USA, 27599

^h^Division of Rheumatology, Department of Medicine, Medical University of South Carolina, Charleston, SC, USA, 29425

^i^Division of Infectious Diseases, Department of Medicine, Medical University of South Carolina, Charleston, SC, USA, 29425

*Corresponding author: Gary Gilkeson, 114 Doughty St., Room 425, Charleston, SC, 29425, Email: [gilkeson@musc.edu](mailto:gilkeson@musc.edu); and Wei Jiang, 173 Ashley Ave., BSB208D, Charleston, SC, 29425, Email: [jianw@musc.edu](mailto:jianw@musc.edu).

**Supplemental figure 1**.


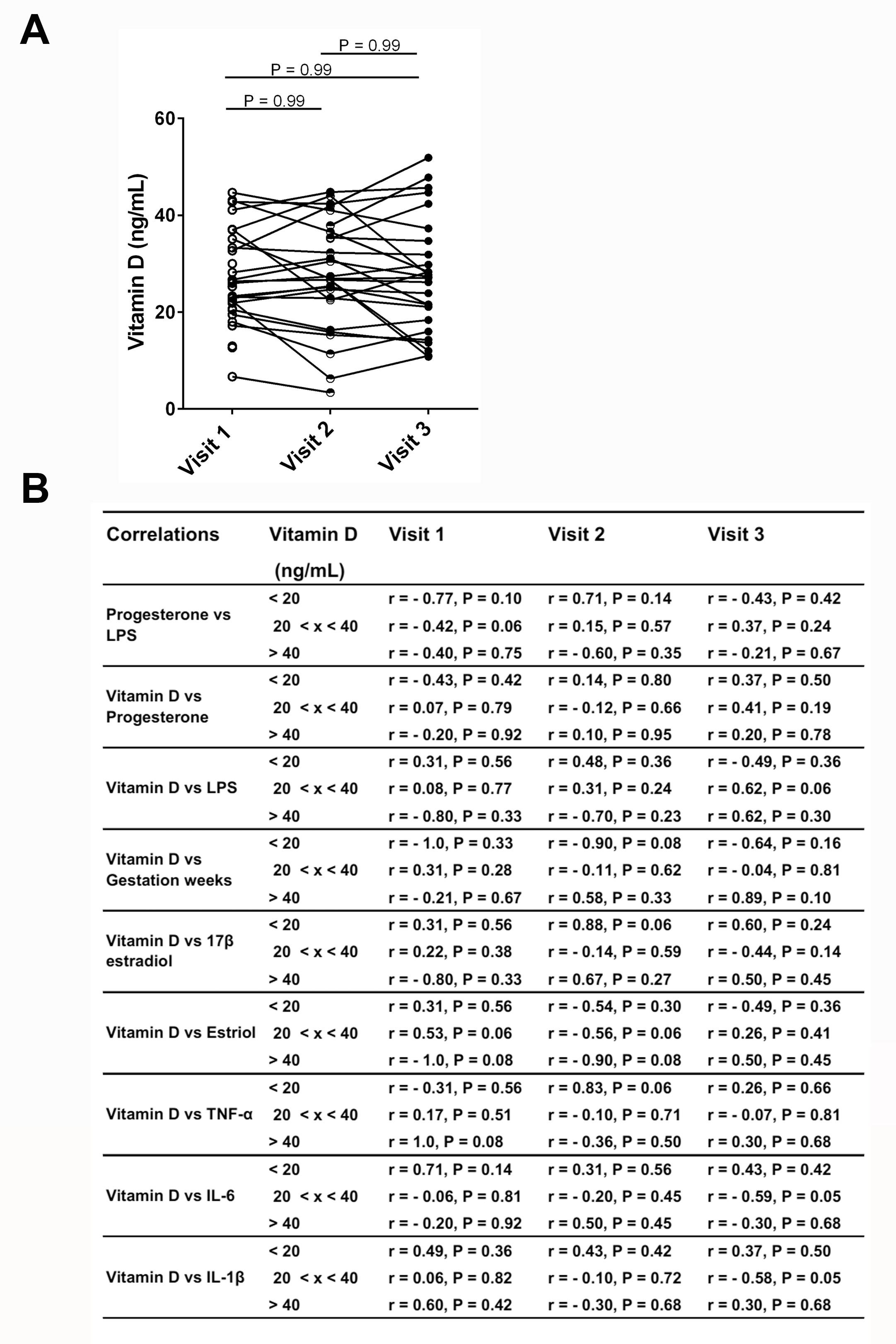


**Supplemental figure 1**. Correlations between progesterone, LPS, 25(OH)D levels, gestation weeks, 17β-estradiol, estriol and cytokines. Plasma levels of circulating 25(OH)D at 8 to 12 weeks gestation (visit 1), 24 to 28 weeks gestation (visit 2) and 6 to 8 weeks postpartum (visit 3) in healthy pregnant women (A). Correlations between progesterone, LPS, 25(OH)D levels, gestation weeks, 17β-estradiol, estriol and cytokines (TNF-α, IL-6, IL-1β) in different subgroups based on the plasma circulating 25(OH)D concentration (< 20 ng/mL is severe deficiency, 20 ng/mL < x < 40 ng/mL is standard deficiency, > 40 ng/mL is a normal level) (B). Spearman correlation test.

**Supplemental figure 2**.


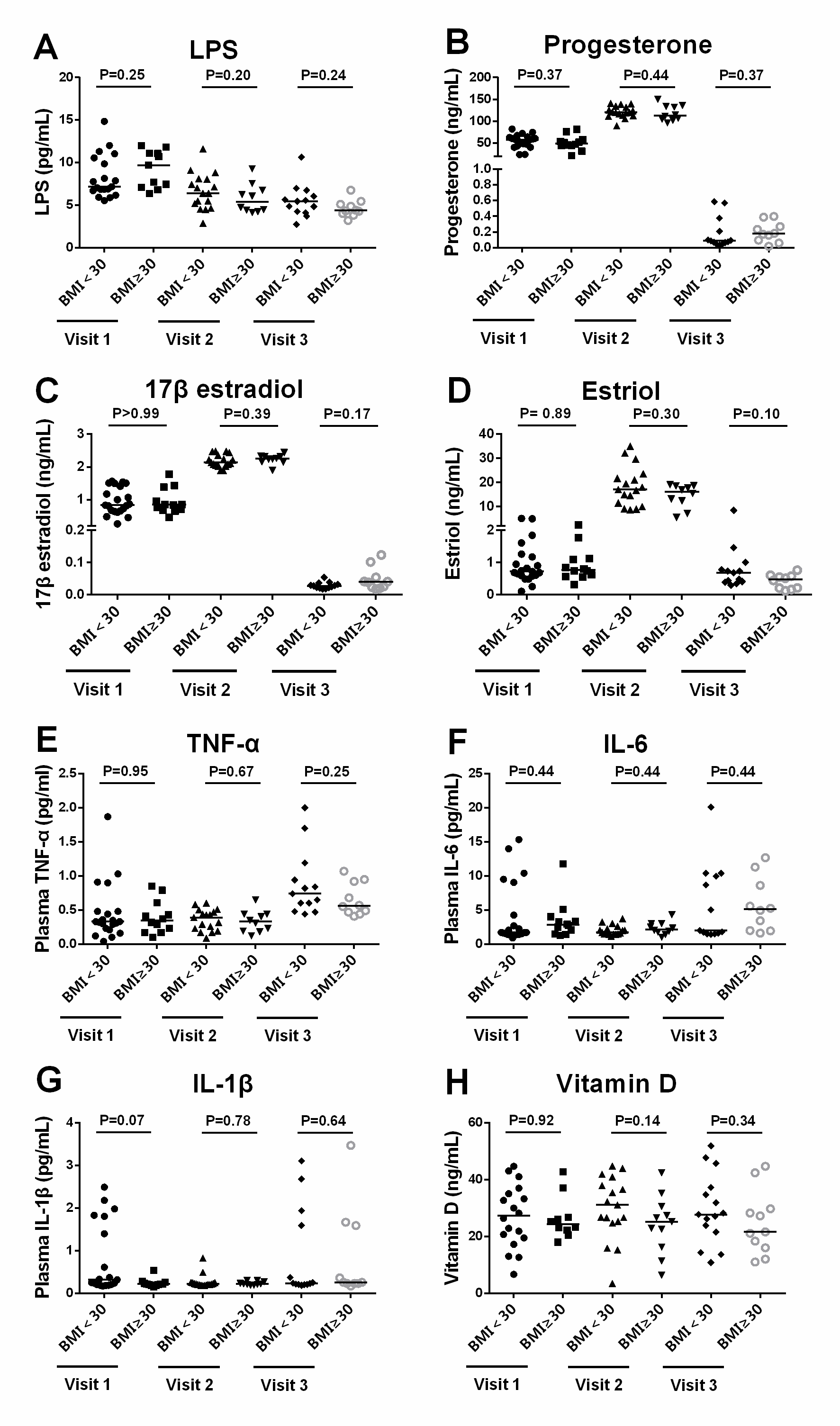


**Supplemental figure 2**. Plasma levels of LPS, 25(OH)D levels, progesterone, 17β estradiol, estriol, TNF-α, IL-6 and IL-1β at 8 to 12 weeks gestation (visit 1), 24 to 28 weeks gestation (visit 2) and 6 to 8 weeks postpartum (visit 3) in two subgroups (BMI <30 and BMI≥30) of healthy pregnant women. One-way ANOVA.

**Supplemental figure 3**.


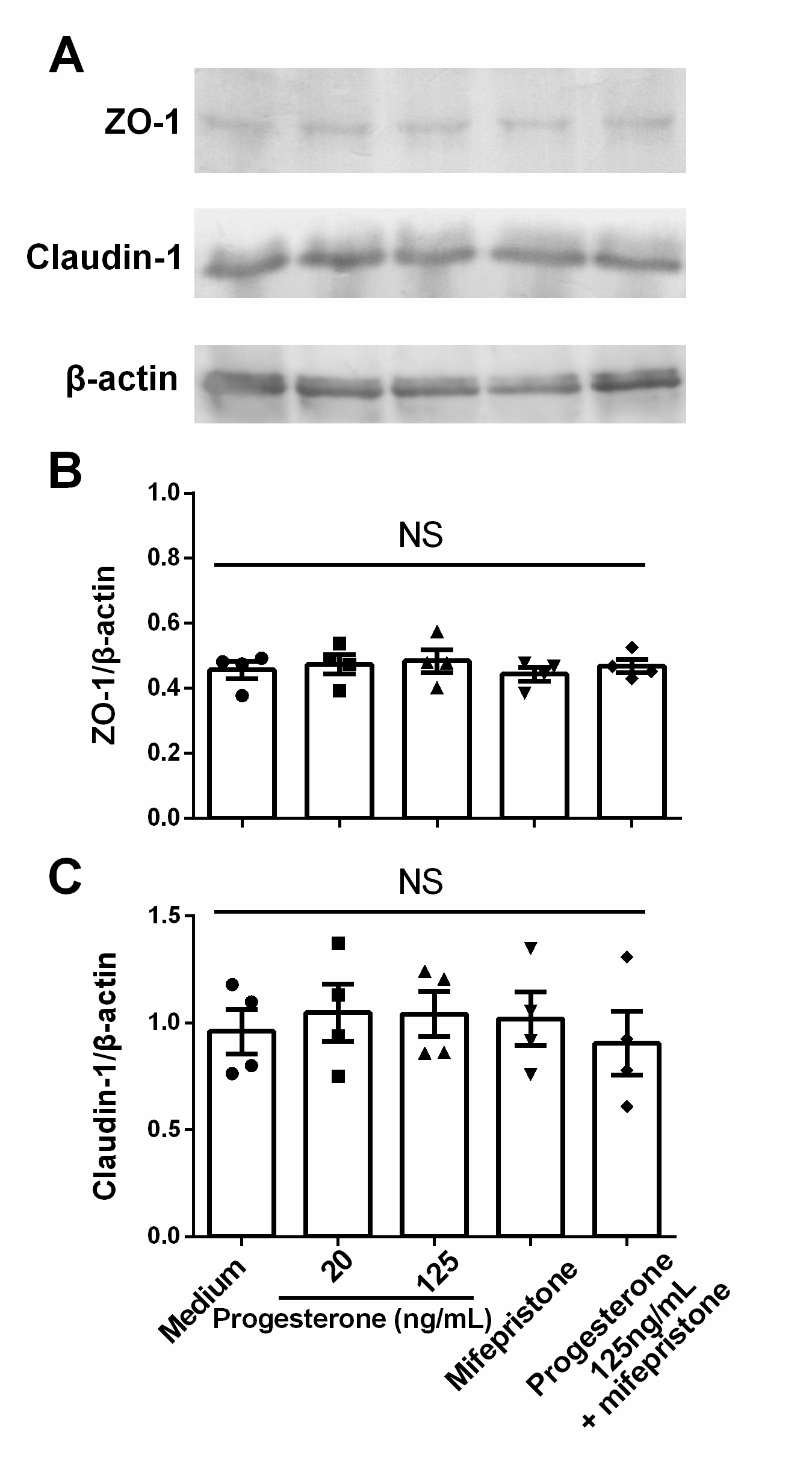


**Supplemental figure 3**. ZO-1 and Claudin-1 expression in Caco-2 cells. Caco-2 cells were treated with media alone or different concentrations of progesterone in the presence or absence of 1 μM mifepristone for 24 h. ZO-1 and Claudin-1 expression were analyzed with western blot (A). Relative expression of ZO-1 (B) and Claudin-1 (C) were quantified to β-actin by ImageJ software. Data are representative of at least three independent experiments. One-way ANOVA.

**Supplemental figure 4**.


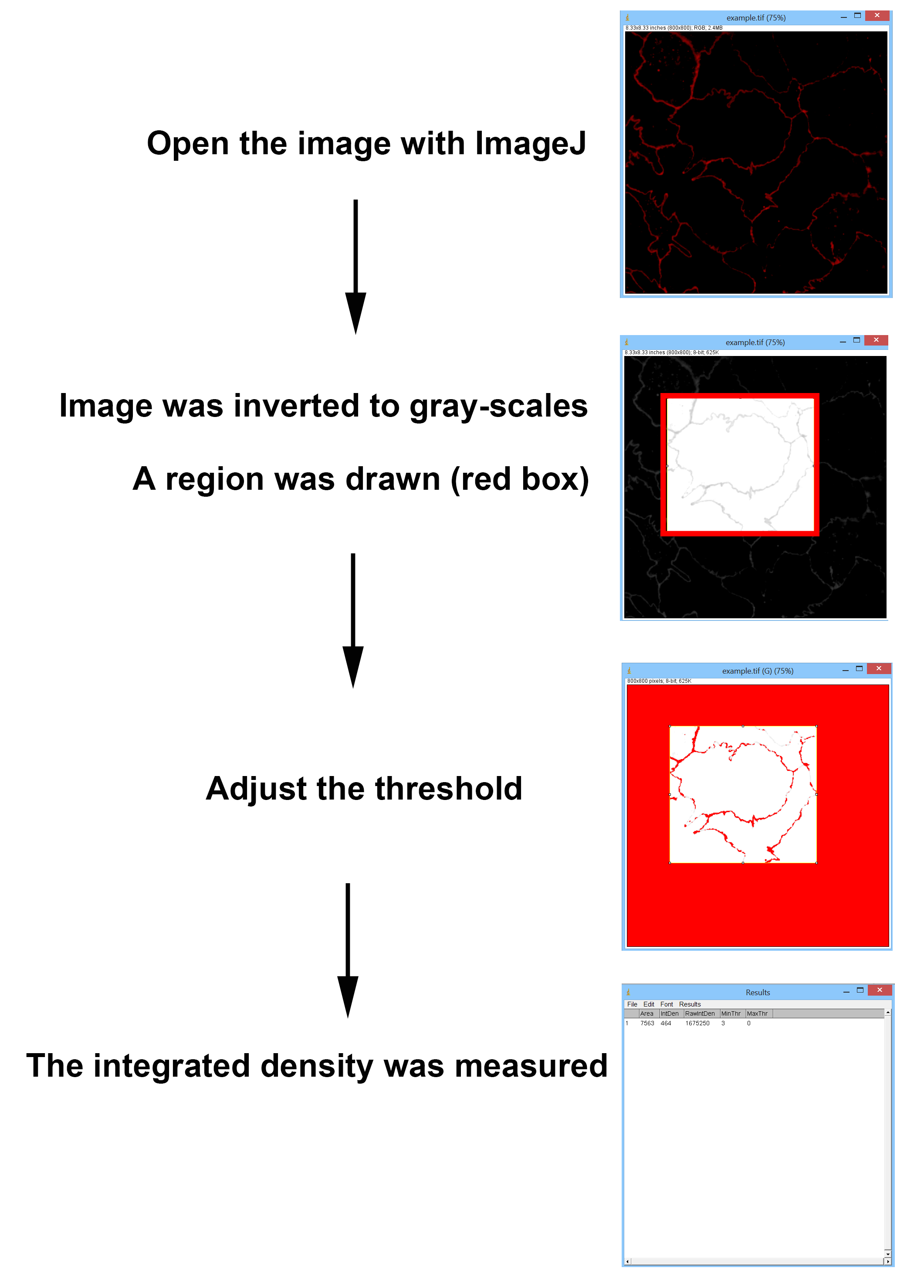


**Supplemental figure 4.** An exemplary image showing how the region of interest was drawn using ImageJ.

**Supplemental figure 5.**


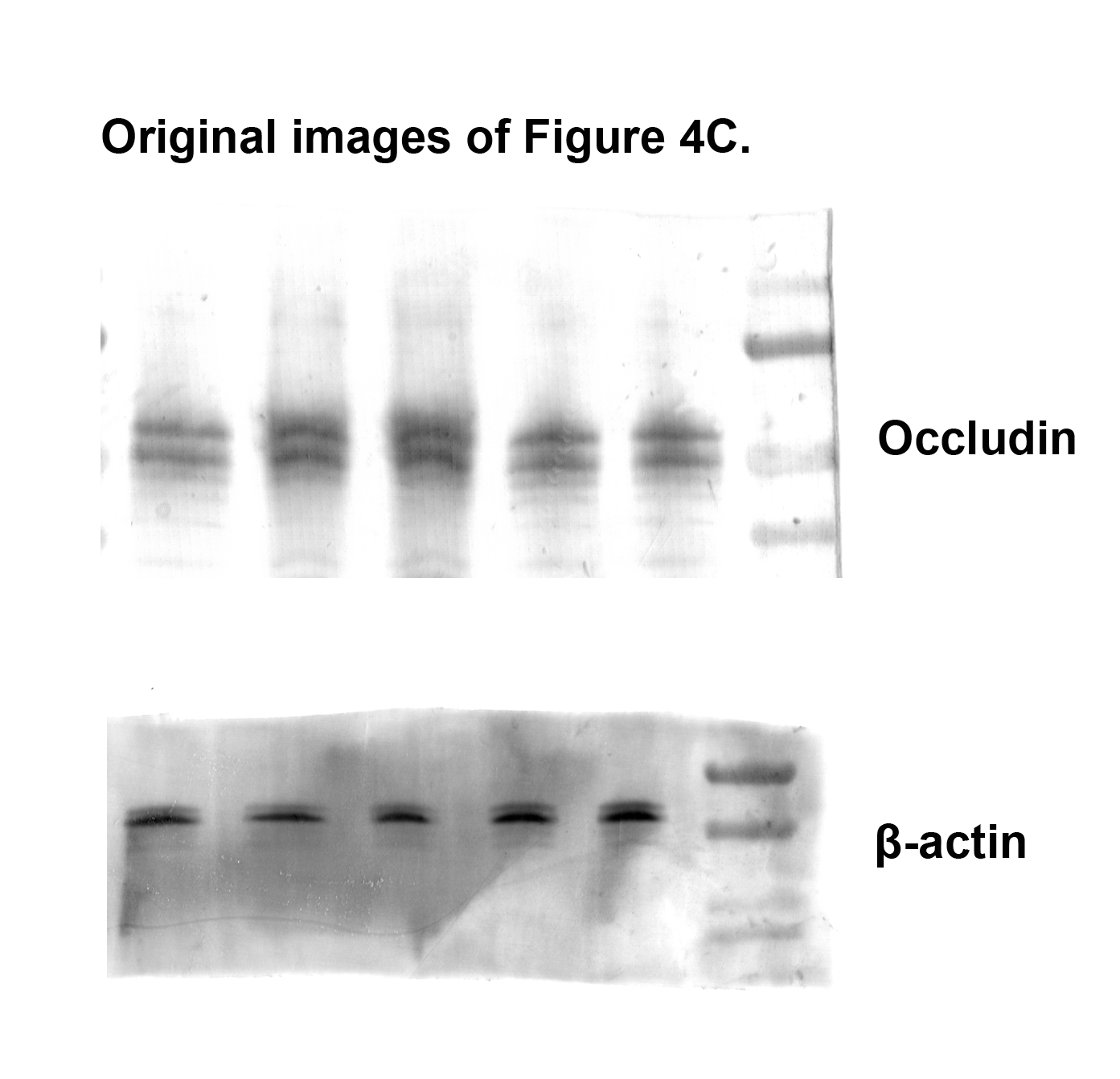


**Supplemental figure 5.** Original images of Figure 4C. Caco-2 cells were pretreated with media alone or different concentrations of progesterone in the presence or absence of 1 μM mifepristone for 24 h. Whole-cell extracts of caco-2 cells were subjected to immunoblot with antibodies against Occludin (upper figure) and β-actin (lower figure).

**Supplemental figure 6.**


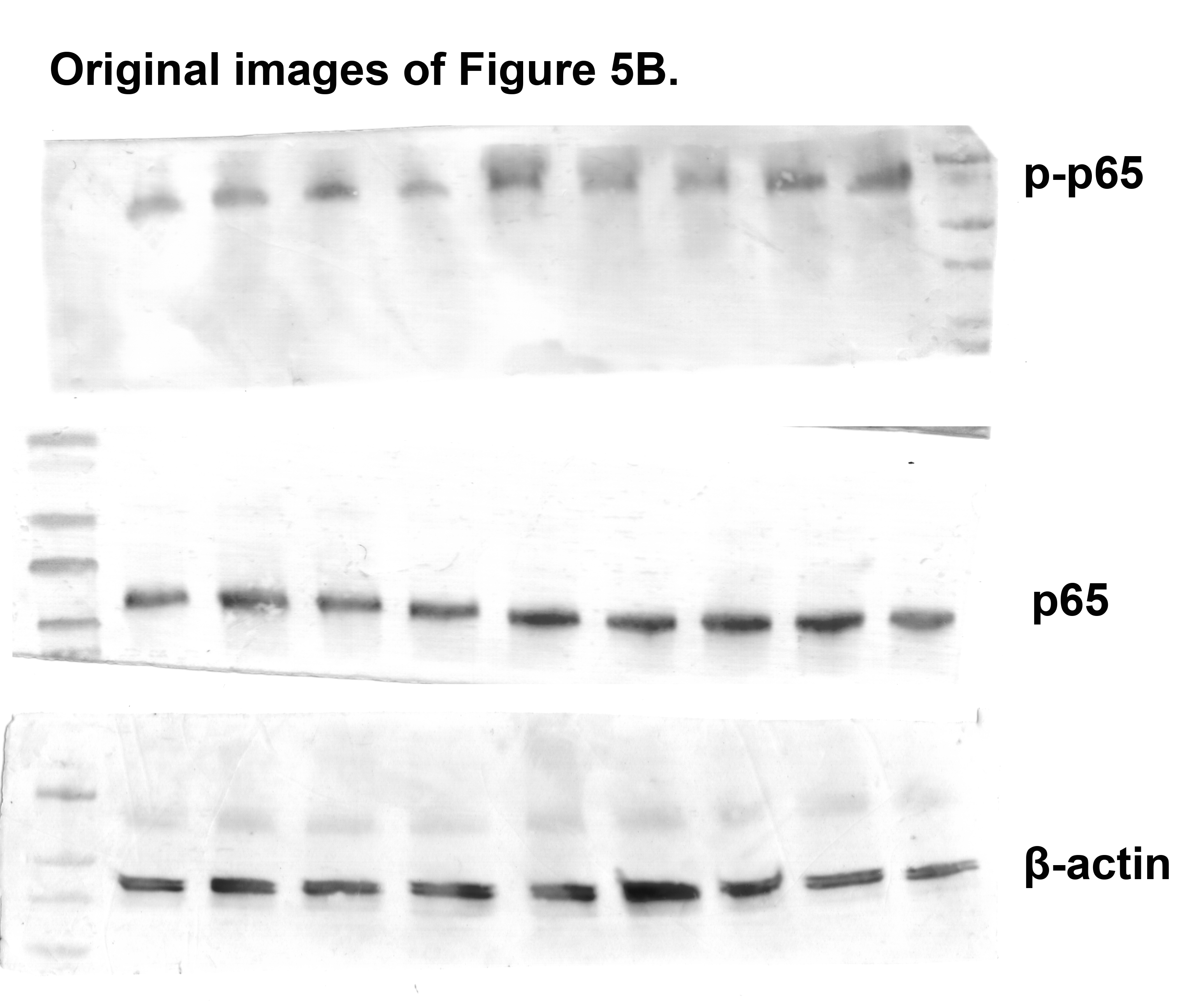


**Supplemental figure 6.** Original images of Figure 5B. Caco-2 cells were pretreated with media alone or different concentrations of progesterone in the presence or absence of 1 μM mifepristone for 24 h, followed by 15 minutes stimulation with 200 ng/mL of LPS. Whole-cell extracts of caco-2 cells were subjected to immunoblot with antibodies against phospho-p65 (p-p65) (upper figure), total-p65 (p65) (middle figure) and β-actin (lower figure).

**Supplemental figure 7.**


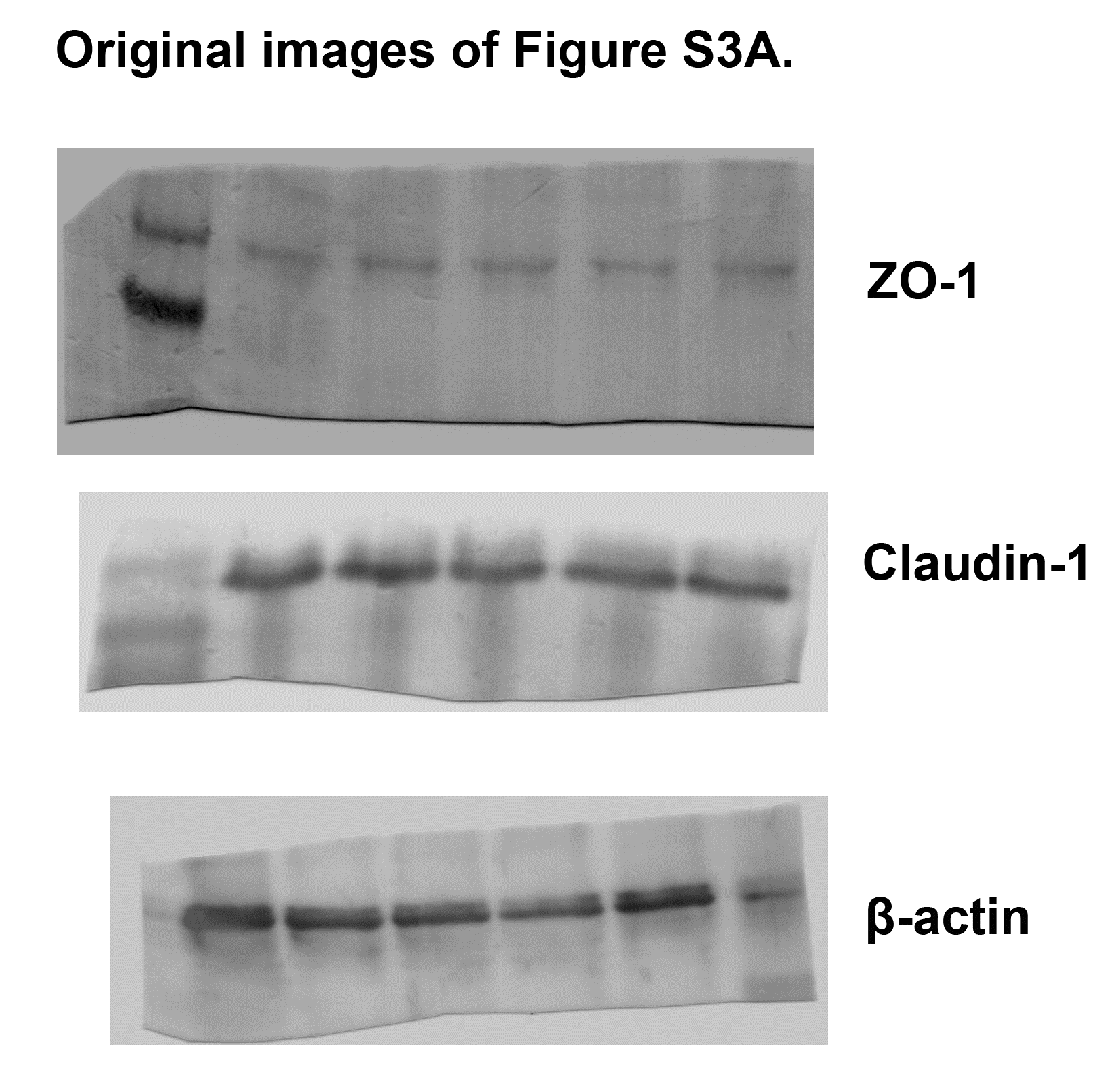


**Supplemental figure 7.** Original images of Figure S3A. Caco-2 cells were pretreated with media alone or different concentrations of progesterone in the presence or absence of 1 μM mifepristone for 24 h. Whole-cell extracts of caco-2 cells were subjected to immunoblot with antibodies against ZO-1 (upper figure), Claudin (middle figure) and β-actin (lower figure).
